# Theoretical Studies on the Mechanism of deNOx Process in Cu–Zn Bimetallic System—Comparison of FAU and MFI Zeolites

**DOI:** 10.3390/molecules27010300

**Published:** 2022-01-04

**Authors:** Izabela Kurzydym, Izabela Czekaj

**Affiliations:** Faculty of Chemical Engineering and Technology, Cracow University of Technology, Warszawska 24, 31-155 Cracow, Poland; wiitch@pk.edu.pl

**Keywords:** zeolites, deNOx, DFT, reaction mechanism, bimetallic catalysts

## Abstract

In the present study we propose a more promising catalyst for the deNOx process to eliminate harmful nitrogen oxides from the environment. The study was performed with a computer calculation using density functional theory (DFT) based on an ab initio method. Two zeolite catalysts, FAU and MFI, were selected with additional Cu–O–Zn bimetallic dimer adsorbed inside the pores of both zeolites. Based on the analysis of preliminary studies, the most probable way of co-adsorption of nitric oxide and ammonia was selected, which became the initial configuration for the reaction mechanism. Two types of mechanisms were proposed: with hydroxyl groups on a bridged position of the dimer or a hydroxyl group on one of the metal atoms of the dimer. Based on the results, it was determined that the FAU zeolite with a bimetallic dimer and an OH group on the zinc atom was the most efficient configuration with a relatively low energy barrier. The real advantage of the Cu–Zn system over FAU and MFI in hydrothermal conditions has been demonstrated in comparison to a conventional Cu–Cu catalyst.

## 1. Introduction

A current problem still related to industrialization is the excessive emission of nitrogen oxides (NOx) [1,2], which pose a serious threat to the environment as well as human health [3,4]. They negatively affect air quality and form photochemical smog and acid rain through atmospheric chemical reactions. Additionally, nitrogen oxide emissions can be precursors to airborne particulate matter (PM_2.5_), which causes damage to human health and negatively affects plant growth [5,6].

One of the best available technologies to reduce nitrogen oxide emissions both in industry and in vehicle exhaust gas treatment systems is the selective catalytic reduction (SCR) process with ammonia as the reducing agent [7,8,9,10]. This technology is widely used, for example, in Diesel engines [11,12]. A catalyst commonly used for this process is V_2_O_5_–WO_3_–TiO_2_ [13,14]. Unfortunately, it has a number of problems connected with a relatively limited working temperature window. Therefore, finding a more efficient and versatile catalyst for the deNOx SCR process has become the interest of many researchers [15,16]. An alternative to the vanadium catalyst is the zeolite catalyst doped with transition metal atoms, especially copper, iron or zinc [17,18,19,20,21].

Recently, various zeolites have been studied for their effectiveness [16,22,23]. Experiments to combine transition metals with zeolites have led to the design of catalysts having higher efficiency, thermal resistance and also increased tolerance to sulfur poisoning [24,25,26]. Additionally, copper has a relatively easy redox cycle during the catalytic reaction, which facilitates the deNOx process [27,28,29]. Zinc also shows interesting catalyst promoting properties for the SCR process [30,31]. Studies have shown that it is an important additive to increase both the temperature window and thermal stability.

Research on bimetallic systems is also of considerable interest [32,33,34,35]. Such catalysts offer the possibility of taking separate advantage of the properties of each metal, which may influence each other to improve catalytic activity.

In the search for more efficient catalysts for the deNOx process, it is also extremely important to understand the mechanism of intermediate reactions that result in the production of harmless nitrogen and water molecules [36,37]. In this aspect, theoretical studies prove to be indispensable, because they allow for a more comprehensive view of the catalytic system and make it possible to test many variants in a relatively short time [38]. The studies have shown that the systems representing metallic dimers with an oxygen atom in the bridge position (M–O–M) have proven to be particularly important [39].

Based on available data, this study proposed a Cu–O–Zn bimetallic system deposited on two zeolites, FAU and MFI, and to check the stability of these systems [40]. After the coadsorption of nitric oxide and ammonia, the mechanisms of the deNOx process on the designed catalysts were proposed. Additionally, variants with partially hydratized dimers were tested, which allowed a comparison of the mechanisms under different conditions. Finally, an analysis of atom ionicity in the systems after coadsorption was also performed, thereby allowing for a more complete understanding of the differences in the mechanisms. All calculations were performed using the density functional theory (DFT) method.

## 2. Results and Discussion

The crystal structure of FAU and MFI was chosen from the Database of Zeolite Structure [41]. The cubic phase of FAU framework type is described by the space group Fd-3m (#227) with lattice constants a = b = c = 24.3450 Å. The crystal unit cell contains 706 atoms. Zeolites with MFI structure crystallize in orthorhombic phase and are characterized by a Pnma (#62) space group with the following lattice parameters: a = 20.090, b = 19.738 and c = 13.142 Å [41]. The crystal unit cell contains 201 atoms.

**Figure 1:**
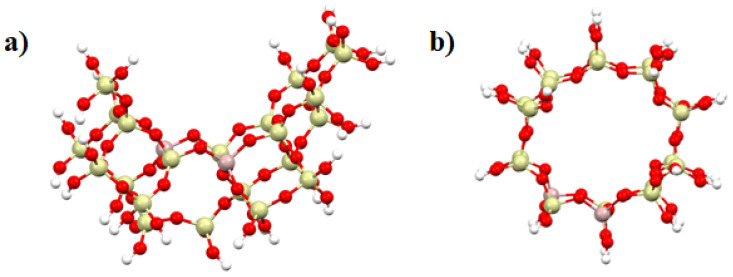
Cluster model of zeolite structures (**a**) FAU (Al_2_Si_22_O_66_H_6_) and (**b**) MFI (Al_2_Si_18_O_53_H_26_), where Si (yellow), Al (pink), O (red) and H (white).

In the calculations, the Al_2_Si_22_O_66_H_6_ cluster (including 24T positions) was used to represent the FAU zeolite, which was formed by cutting a fragment with an active site: replacing to silicon atoms with two aluminum ones (Figure 1a). The same procedure was used for the MFI zeolite. The Al_2_Si_18_O_53_H_26_ cluster (including 20T positions) containing the entire zeolite pore was cut from the crystal structure (Figure 1b). The positions of Al atoms was chosen according to previous studies [42], with distance between Al–Al equal to 5 Å. In each cluster, dangling bonds problem and cluster neutrality were achieved by saturating the peripheral oxygen atoms with hydrogen atoms at the standard OH distance (0.97 Å) in the direction of the respective broken Si–O bonds. The central part of the cluster, including the Al centers and neighboring Si and O, were optimized.

**Figure 2:**
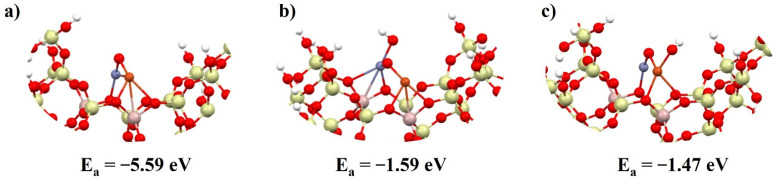
Cluster model of FAU zeolite structure with deposited bimetallic dimer (**a**) copper–zinc dimer, (**b**) copper–zinc dimer with hydroxyl group adsorbed on zinc, and (**c**) copper–zinc dimer with hydroxyl group adsorbed on copper, with adsorption energy below the structure. Zn (blue), Cu (orange).

**Figure 3:**
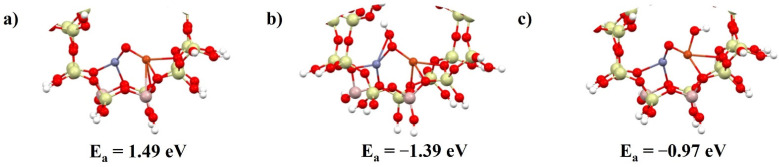
Cluster model of MFI zeolite structure with deposited bimetallic dimer (**a**) copper–zinc dimer, (**b**) copper–zinc dimer with hydroxyl group adsorbed on zinc, and (**c**) copper–zinc dimer with hydroxyl group adsorbed on copper, with adsorption energy below the structure.

In the next step, a Cu–O–Zn bimetallic system was adsorbed near the aluminum atoms (active site) in both FAU and MFI zeolite (Figure 2 and Figure 3). Additionally, structures with one OH group on zinc or copper were also designed. The partial hydration simulated the water molecules in the reaction medium (Figure 2b,c and Figure 3b,c). As can be seen, definitely a more durable system with metallic dimer was formed on the FAU zeolite. In the case of the MFI zeolite, energy (1.49 eV) had to be supplied to deposit the dimer inside the pore. On the other hand, the following adsorption of OH molecule proceeded in an exothermic way with similar energies for both FAU and MFI: between −0.97 and −1.59 eV.

Despite the similar energies, the adsorption of the hydroxyl group onto zinc was more energetically favorable. In the case of the FAU zeolite, the OH group attached only to zinc, whereas in the MFI zeolite, an oxygen–oxygen bridging bond was also formed. This was explained by the lower ionicity of the oxygen bridge atoms in the bimetallic system depending on which zeolite was deposited onto it (Figure S1).

The next step was to carry out preliminary adsorption and coadsorption to initiate the deNOx mechanism, which was based on previous studies performed on the FAU and MFI zeolite with a deposited Cu–O-Cu dimer [43].

Two initial steps were considered for the non-hydratized bimetallic dimer: NO adsorption and coadsorption of NO and NH_3_ (Figures S2 and S3). The adsorption energy was counted for each structure. Structures releasing more energy were selected for further analysis because the probability of forming these types of structures was higher.

Figure S2 shows that, for both zeolite FAU and MFI, the most stable system was formed when NO was adsorbed onto the bridging oxygen. Interestingly, in the case of the MFI zeolite there was no possibility that nitric oxide would bind to zinc. NO can be bound only by bridge oxygen. Based on these studies, NO systems adsorbed onto the bridging oxygen were selected for the coadsorption of ammonia. After carrying out the coadsorption process in different variants (Figure S3), the systems with the lowest energy were selected for further analysis. In the case of the FAU zeolite, NO adsorbs both onto bridging oxygen and copper, while NH_3_ adsorbs onto zinc. In contrast, in MFI, NH_3_ adsorbs onto copper and NO onto zinc.

For systems with an adsorbed OH group on one of the metal atoms in the dimer, the procedure for selecting the mechanism structure was simpler because the first step directly involved coadsorption (Figures S4 and S5). Here, again, the structures which were more stable and released more energy into the environment during their formation were chosen. For the FAU zeolite with an OH group on Cu in the dimer, a structure with NO adsorbed onto bridging oxygen was chosen, while NH_3_ was adsorbed onto zinc. In this case, the MFI zeolite with an OH group on Cu showed the same adsorption mode; however, a partial decomposition of the ammonia molecule had already occurred in the first step, and one of the hydrogen atoms detached from the ammonia and adsorbed onto the nitric oxide oxygen, which also formed a bond with the copper in the dimer. In the case of zeolites with an OH group on zinc for FAU, a system analogous to the previous one was chosen—NO adsorbed onto bridged oxygen and NH_3_ onto copper. For the MFI zeolite, adsorption proceeded in the same way, but NO also formed an additional bond with copper. In this case, however, there was no partial decomposition of NH_3_.

**Figure 4:**
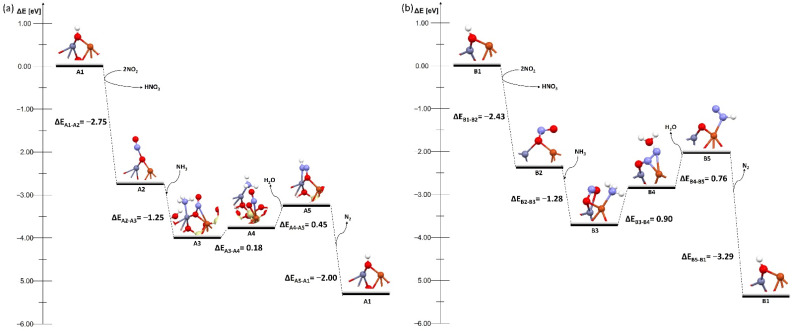
Energy diagram of proposed mechanism of deNOx in the Cu–O–Zn dimer supported on (**a**) bimetallic dimer on FAU and (**b**) bimetallic dimer on MFI with bridged OH group on both zeolites.

**Figure 5:**
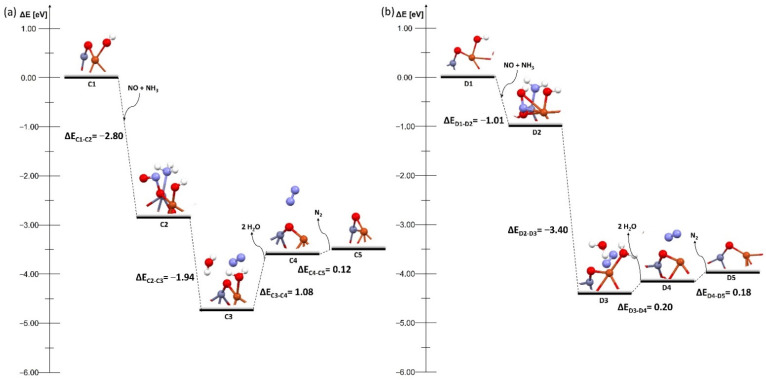
Energy diagram of proposed mechanism of deNOx in the Cu–O–Zn dimer supported on (**a**) bimetallic dimer on FAU and (**b**) bimetallic dimer on MFI with OH group on Cu on both zeolites.

**Figure 6:**
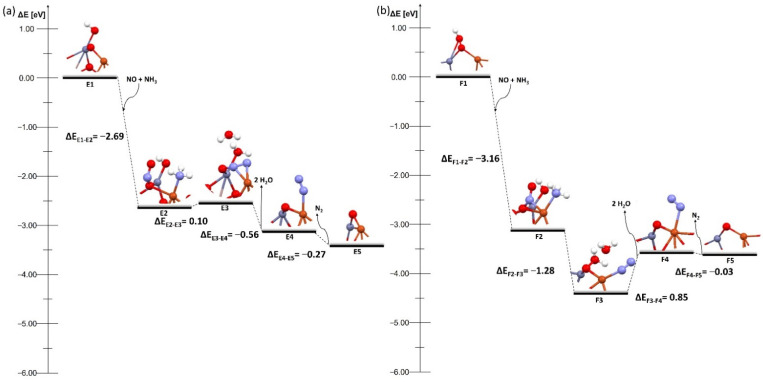
Energy diagram of proposed mechanism of deNOx in the Cu–O–Zn dimer supported on (**a**) bimetallic dimer on FAU and (**b**) bimetallic dimer on MFI with OH group on Zn on both zeolites.

Based on these preliminary findings, reaction mechanisms were proposed for each structure type (Figure 4, Figure 5 and Figure 6). Based also on previous detailed mechanism studies by Bendrich et al., systems with different ways of partial hydratized dimer were proposed [44]. The re-oxidation step for copper centers, presented in detail in the literature [45], is not discussed here; instead, we concentrated on reactions barriers for deNOx in the Cu–O–Zn dimer.

The first pathway is represented by a system with a bridged OH group (Figure 4). The process occurs in five steps. First (Figure 4 A2), after the reaction of the catalyst with two nitrogen dioxide molecules, one nitric acid molecule was formed and an NO molecule was adsorbed on the catalyst surface. The next step was the adsorption of NH_3_ (Figure 4 A3). After adsorption, the system was transformed to form a water molecule (Figure 4 A4), when further desorption took place, and a nitrogen molecule was formed (Figure 4 A5), which broke away from the surface, and the system returned to its initial configuration. In both zeolites the process proceeded in the same way and an energy barrier was also in the same places. The barrier appeared at the moment the system transformed into a water molecule during its desorption. In the case of the MFI zeolite these barriers were higher (0.90 and 0.76 eV, Figure 4 B4 and B5, respectively) than in case of the FAU zeolite (0.18 and 0.45 eV, Figure 4 B4 and B5). The barriers on the bimetallic dimer on the FAU were lower than for conventional Cu–Cu dimers (0.65 and 0.41 eV, Figure S6 A4 and A5). Thus it could be concluded that the bimetallic dimer deposited onto the FAU zeolite with an OH group on bridged oxygen was a more efficient catalytic system than the one on the MFI zeolite. However, the reaction was more effective on the conventional copper dimer on the MFI and proceeded without barrier (Figure S6b).

The next two mechanisms occur on systems with an OH group on one of the metals in the dimer (Figure 5 and Figure 6), and they proceed in exactly the same way. In the first step NO and NH_3_ are coadsorbed, and then the transformation of the system to two water molecules takes place. In the next stage, these molecules desorb, and a nitrogen molecule is formed, which then desorbs from the surface. The final step is the regeneration of the system with one OH group.

Several changes were observed while analyzing these mechanisms. First, there was a shift of the energy barrier in one stage of the process compared to systems with an OH group on the bridged oxygen. The barrier in the systems with the OH group on the copper atom did not occur at the moment of the transformation of the system but during the desorption of water molecules and the nitrogen molecule. The barriers are 1.08 and 0.12 eV for the FAU zeolite (Figure 5 C4 and C5) and 0.20 and 0.18 eV for the MFI zeolite (Figure 5 D4 and D5), respectively. The systems with the OH group on zinc broke this pattern. In the FAU zeolite, the energy barrier occurred only at the stage of the transformation of the system without the adsorption or desorption of any molecules, and was relatively low—0.1 eV (Figure 6 E3), whereas for the MFI zeolite the barrier was present only during the desorption of water molecules and amounted to 0.85 eV (Figure 6 F4). The barriers on the bimetallic dimer on FAU and MFI were lower than in the case of conventional Cu–Cu dimers on FAU and MFI (0.81 and 1.07 eV, Figure S7 C4 and D4). It could be clearly seen here that the hydration mode of the system significantly affected the deNOx process.

The process proceeded most efficiently for systems in which the OH group was located on zinc because the energy barrier was present at only one stage of the process (Figure 6). In addition, a catalyst with a dimer deposited on the FAU zeolite was particularly promising (Figure 6a) as the energy barrier of the transformation stage of the system was very low, so the deNOx process could proceed without additional expenditure related to the temperature increase. The results of our study were also confirmed by the experimental study carried out by Xu et al. [30] who found that the addition of Zn increased the hydrothermal stability, especially when the Cu–O–Zn dimers are formed. They also found that zinc helped to stabilize the zeolite framework and prevent the migration of Cu^2+^ ions, which was essential during deNOx processes.

**Figure 7:**
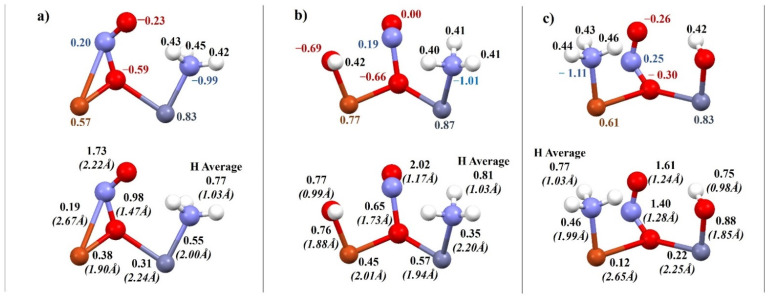
Charge distribution (above), bond order and length—bracketed (below) for coadsorption of NO and NH_3_ on metallic Cu–Zn dimers in FAU: (**a**) Cu–Zn bimetallic dimer, (**b**) Cu–Zn bimetallic dimer with OH group on Cu, (**c**) Cu–Zn bimetallic dimer with OH group on Zn.

**Figure 8:**
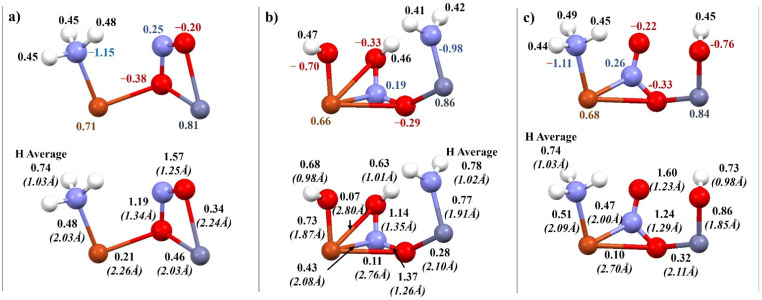
Charge distribution (above), bond order and length—bracketed (below) for coadsorption of NO and NH3 on metallic Cu–Zn dimers in MFI: (**a**) Cu–Zn bimetallic dimer, (**b**) Cu–Zn bimetallic dimer with OH group on Cu, (**c**) Cu–Zn bimetallic dimer with OH group on Zn.

The charge distribution, bond order and bond length of the systems after coadsorption for the FAU (Figure 7) and the MFI zeolite (Figure 8) were also performed to understand the deNOx process better. When analyzing the charge on the atoms, some differences were noticed (Figure 7). In the FAU zeolite with an OH group on the zinc atom, the bridged oxygen charge was twice as high (Figure 7c) as that of the other FAU systems (Figure 7a,b). In addition, the nitrogen–bridge oxygen bond order was stronger for FAU than for MFI zeolite, which resulted in an energy barrier to the formation and desorption of N_2_ in the reaction mechanism. In contrast, for the bimetallic dimer system without OH groups, the energy barrier occurred due to the strong binding of ammonia to the metal (nitrogen–zinc bond order 0.55 for MFI and 0.48 for FAU; Figure 7a).

While analyzing the results for the MFI zeolite, differences were noticed (Figure 8). In Figure 8b it was observed that the coadsorption step ran differently for the system that had an OH group on copper. NH_3_ decomposed and one of the hydrogen atoms attached to the oxygen in the nitric oxide. This resulted in the extended nitrogen–oxygen bond in nitric oxide and in a further step promoted the formation of water and N_2_ molecules.

## 3. Materials and Methods

The electronic structure of all clusters was calculated by ab initio density functional theory (DFT) methods (StoBe program, [46]) using the non-local generalized gradient corrected functionals according to Perdew, Burke, and Ernzerhof (RPBE) [47,48], to account for the electron exchange and correlation. All Kohn–Sham orbitals were represented by linear combinations of atomic orbitals (LCAOs) using contracted Gaussian basis sets for the atoms [49]. A detailed analysis of the electronic structure of the clusters was carried out using Mulliken populations [50] and Mayer bond order indices [51,52].

Double valence zeta polarization (DZVP) functional bases were used for orbital basis sets Cu and Zn (63321/531/311), Si, Al (6321/521/1), O, N (621/41/1) and H (41). Additionally, auxiliary functional bases were used to adjust the density electron and exchange potential of the correlation of individual atoms: Si and Al (5,4;5,4), Cu and Zn (5,5;5,5), O, N (4,3;4,3) and H (4,0;4,0).

All structures were visualized using Mercury software [53].

The calculations took into account the structures with the lowest energy, and for each structure all probable multiplets were calculated and all the structures were allowed to relax in each electronic state.

## 4. Conclusions

To summarize the analysis, it can be concluded that:

1. The bimetallic dimer selected in this study, Cu–Zn, showed high stability in the FAU zeolite, while their adsorption on the MFI zeolite proceeded with an energy barrier. The deNOx reaction mechanism was the same regardless of zeolite type.
2. All NO and NH_3_ coadsorption variants occurred spontaneously with energy release.
3. To propose the mechanism of the deNOx process, the most stable structures (emitting the highest amount of energy) were chosen because of the most probable formation of these systems inside the zeolite frame.
4. Two types of mechanism were proposed depending on the type of dimer hydration. It was shown that energy barriers occurred between different stages depending on the type of hydration.
5. The most efficient reaction mechanism was represented by the FAU zeolite with bimetallic Cu–Zn dimer and hydrated zinc center because during the process the energy barrier appeared only in one stage and was relatively low.
6. The real advantage of the Cu–Zn system over FAU and MFI in hydrothermal conditions was demonstrated in comparison to a conventional Cu–Cu catalyst.
7. This study provided a good basis for comparison with experimental results to confirm the theoretically obtained adsorption mechanisms.

## Data Availability

Data is contained within the article or Supplementary Materials.

## Supplementary Materials

The following are available online. Figure S1: Charge distribution for metallic Cu–Zn dimers in FAU: (a) Cu–Zn bimetallic dimer, (b) Cu–Zn bimetallic dimer with OH group on Cu, (c) Cu–Zn bimetallic dimer with OH group on Zn and in MFI (d) Cu–Zn bimetallic dimer, (e) Cu–Zn bimetallic dimer with OH group on Cu, (f) Cu–Zn bimetallic dimer with OH group on Zn; Figure S2: Adsorption of NO on Cu–O–Zn dimer in FAU zeolite: (a) NO on Cu in dimer, (b) NO on Zn in dimer, (c) NO on oxygen bridge in dimer; and in MFI zeolite: (d) NO on Cu in dimer (e) NO on oxygen bridge in dimer. Energies of adsorption below the structure; Figure S3: Coadsorption of NO and NH_3_ on Cu–O–Zn dimer in FAU zeolite: (a) NH_3_ on Cu in dimer, (b) NH_3_ on Zn in dimer; and in MFI zeolite: (c) NO on Cu in dimer (d) NO on oxygen bridge in dimer. Energies of adsorption below the structure; Figure S4: Coadsorption of NO and NH_3_ on Cu–O–Zn dimer with OH group on Cu in FAU zeolite: (a) NH_3_ on Cu in dimer, (b) NH_3_ on Zn in dimer; and in MFI zeolite: (c) NO on Cu in dimer (d) NO on oxygen bridge in dimer. Energies of adsorption below the structure; Figure S5: Coadsorption of NO and NH_3_ on Cu–O–Zn dimer with OH group on Zn in FAU zeolite: (a) NH_3_ on Cu in dimer, (b) NH_3_ on Zn in dimer; and in MFI zeolite: (c) NO on Cu in dimer (d) NO on oxygen bridge in dimer. Energies of adsorption below the structure. Figure S6: Energy diagram of proposed mechanism of deNOx in the Cu–O-Cu dimer supported on (a) copper dimer on FAU and (b) copper dimer on MFI with bridged OH group on both zeolites. Figure S7: Energy diagram of proposed mechanism of deNOx in the Cu–O–Zn dimer supported on (a) copper dimer on FAU and (b) copper dimer on MFI with OH group on Cu on both zeolites. Description of the formula for calculating the energy of the various systems and the differences between the stages of the mechanism. Table S1 Energies for different structures and multiplicities.

## Sample Availability

Samples of the compounds used in this studies are available from the authors.
