# Supplementary material for: Theoretical Studies on the Mechanism of deNOx Process in Cu–Zn Bimetallic System—Comparison of FAU and MFI Zeolites
Source: Molecules. 2022 Jan 4;27(1):300. doi: 10.3390/molecules27010300 (PMC8746640; doi:10.3390/molecules27010300)
Supplement: Supplementary file 1 [file molecules-27-00300-s001.zip › molecules-1505647-supplementary.pdf]

Supplementary Informations of Article

Theoretical studies on the mechanism of deNOx process in Cu-Zn bimetallic system - comparison of FAU and MFI zeolites

Izabela Kurzydym [1], and Izabela Czekaj [1,*]

[1] Faculty of Chemical Engineering and Technology, Cracow University of Technology, Warszawska 24, 31-155 Kraków, Poland; wiitch@pk.edu.pl

* Correspondence: izabela.czekaj@pk.edu.pl

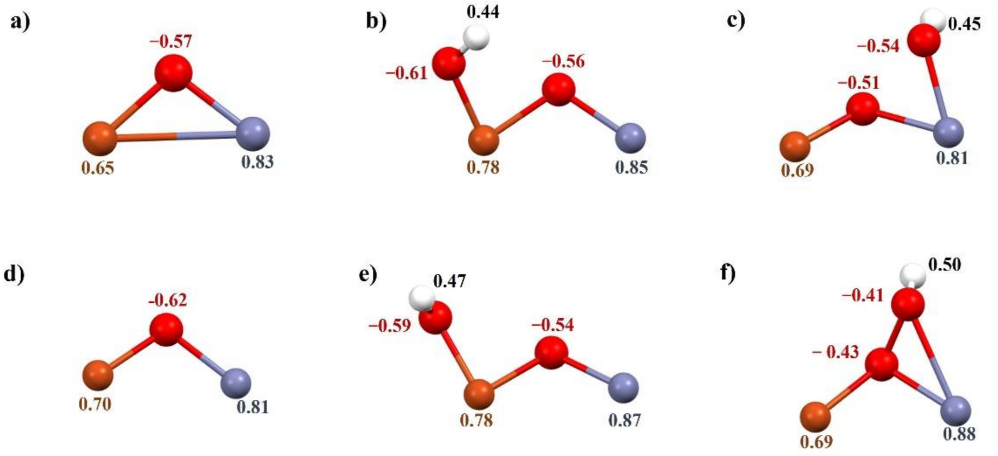

Figure S1. Charge distribution for metallic Cu-Zn dimers in FAU: a) Cu-Zn bimetallic dimer, b) Cu-Zn bimetallic dimer with OH group on Cu, c) Cu-Zn bimetallic dimer with OH group on Zn and in MFI d) Cu-Zn bimetallic dimer, e) Cu-Zn bimetallic dimer with OH group on Cu, f) Cu-Zn bimetallic dimer with OH group on Zn.

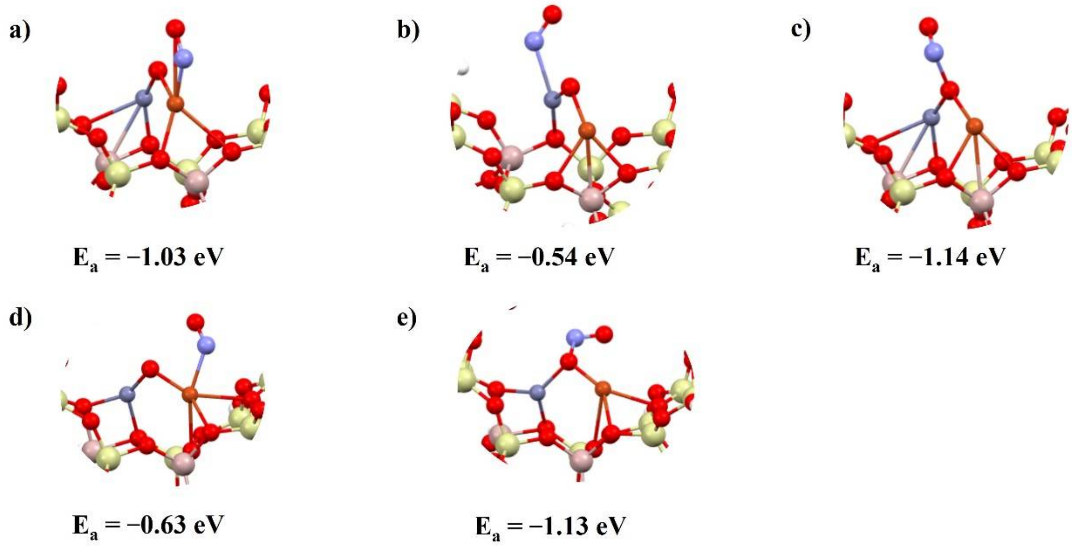

Figure S2. Adsorption of NO on Cu-O-Zn dimer in zeolite FAU: a) NO on Cu in dimer, b) NO on Zn in dimer, c) NO on oxygen bridge in dimer; and in zeolite MFI: d) NO on Cu in dimer e) NO on oxygen bridge in dimer. Energies of adsorption below structure.

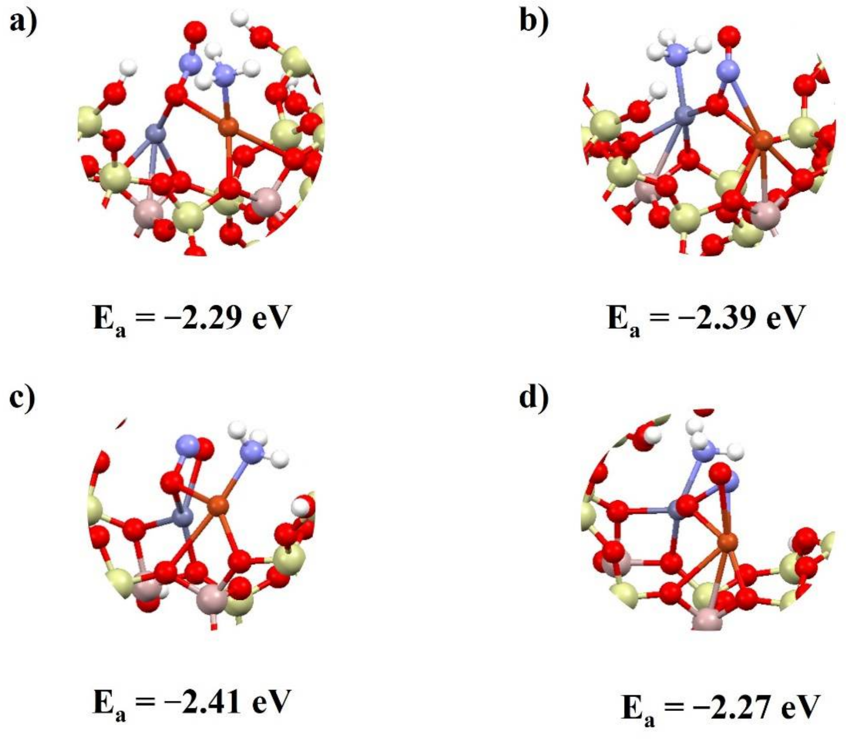

Figure S3. Coadsorption of NO and NH_3 on Cu-O-Zn dimer in zeolite FAU: a) NH_3 on Cu in dimer, b) NH_3 on Zn in dimer; and in zeolite MFI: c) NO on Cu in dimer d) NO on oxygen bridge in dimer. Energies of adsorption below structure.

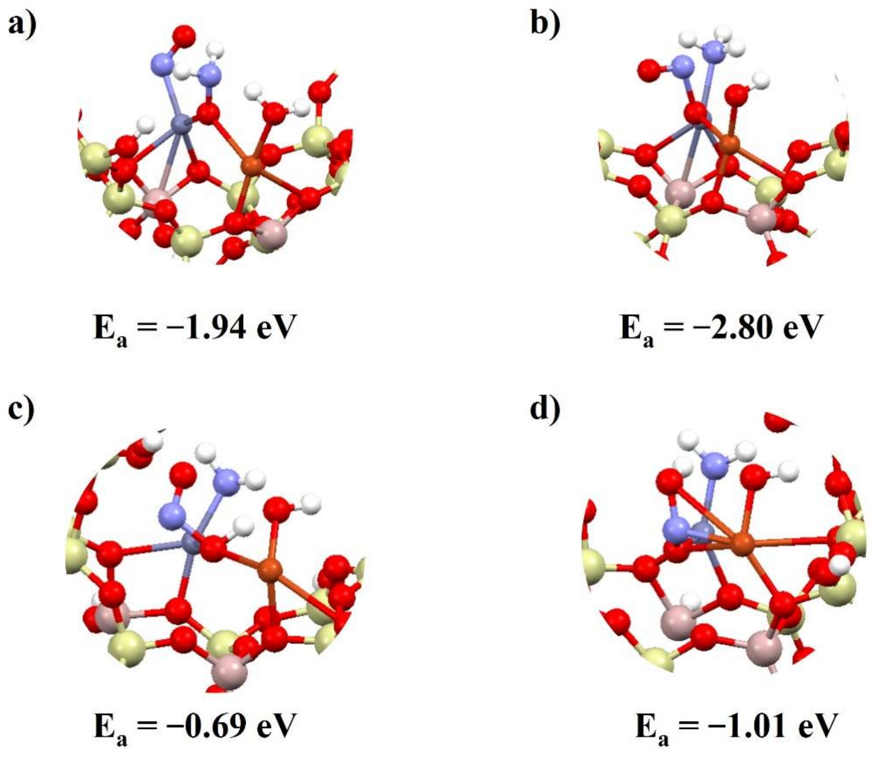

Figure S4. Coadsorption of NO and NH_3 on Cu-O-Zn dimer with OH group on Cu in zeolite FAU: a) NH_3 on Cu in dimer, b) NH_3 on Zn in dimer; and in zeolite MFI: c) NO on Cu in dimer d) NO on oxygen bridge in dimer. Energies of adsorption below structure.

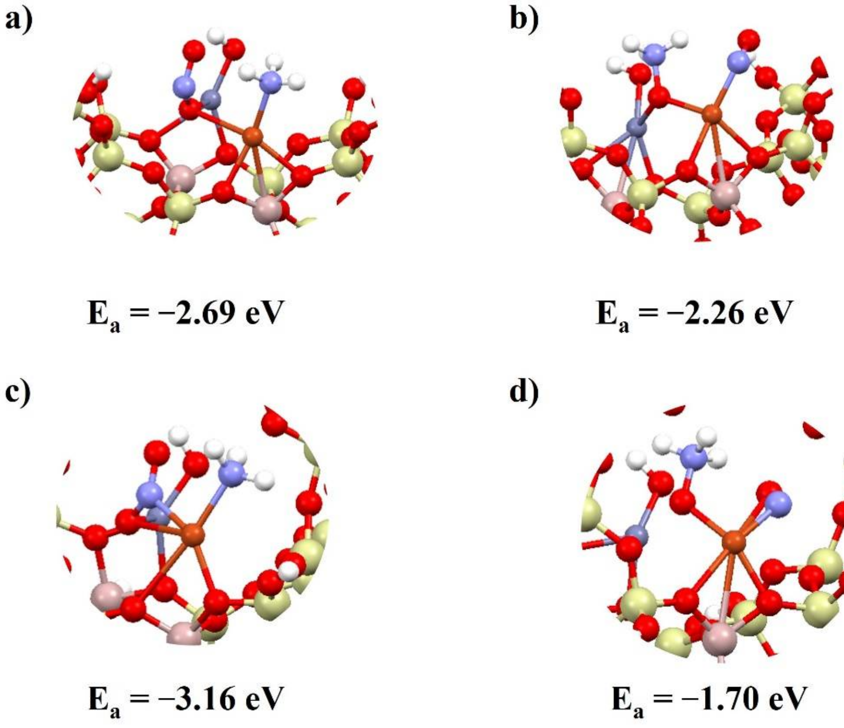

Figure S5. Coadsorption of NO and NH_3 on Cu-O-Zn dimer with OH group on Zn in zeolite FAU: a) NH_3 on Cu in dimer, b) NH_3 on Zn in dimer; and in zeolite MFI: c) NO on Cu in dimer d) NO on oxygen bridge in dimer. Energies of adsorption below structure.

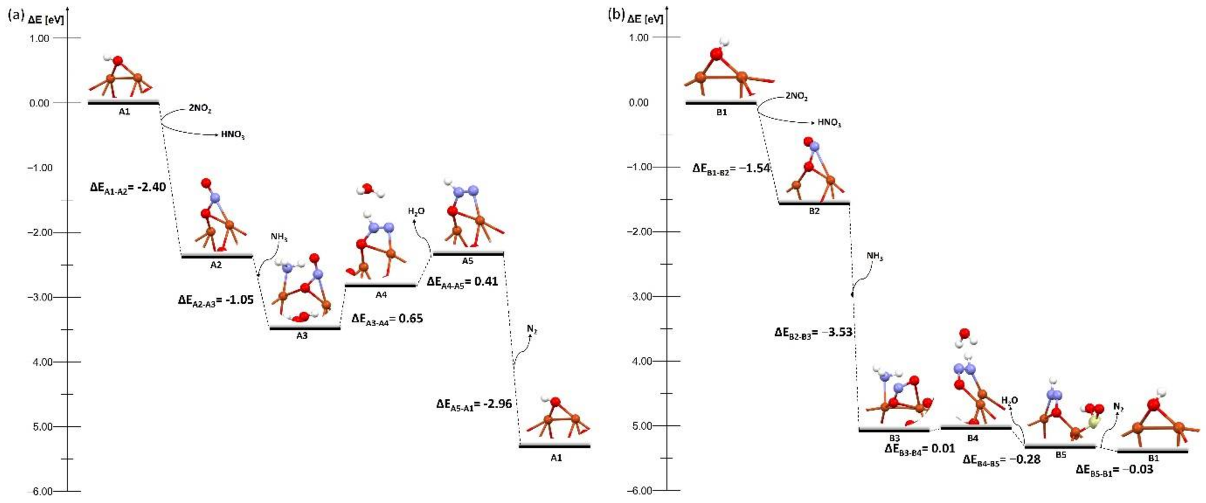

Figure S6. Energy diagram of proposed mechanism of deNOx in the Cu-O-Cu dimer supported on a) copper dimer on FAU and b) copper dimer on MFI with bridged OH group on both zeolites.

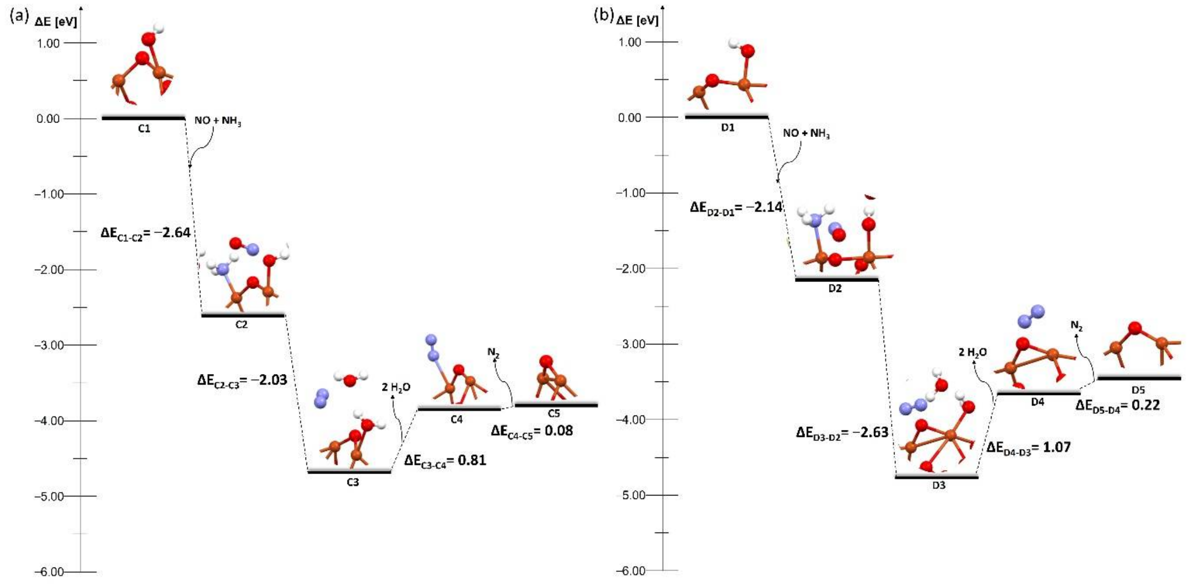

Figure S7. Energy diagram of proposed mechanism of deNOx in the Cu-O-Zn dimer supported on a) copper dimer on FAU and b) copper dimer on MFI with OH group on Cu on both zeolites.

The adsorption energy of individual structures was calculated according to the formula below.

The adsorption energies of the adsorbates on the cluster were calculated as follows:

$$E_a(\text{adsorbate/cluster}) = E_{tot}(\text{adsorbate/cluster}) - E_{tot}(\text{cluster}) - E_{tot}(\text{adsorbate}),$$

where E_{tot}(adsorbate/ cluster) is the total energy of the adsorbate/cluster surface complex, E_{tot}(cluster) and E_{tot}(adsorbate) are the total energies of pure cluster and the adsorbate, respectively.

Energy difference between stages in mechanism with bridged OH group were calculated as follows:

1. Energy difference between stage A2 and A1 (same for B diagram):

$$E_{diff} = E_{A2} – E_{A1} – 2E_{NO2} + E_{HNO3} \text{ [eV]}$$

2. Energy difference between stage A3 and A2 (same for B diagram):

$$E_{diff} = E_{A3} – E_{A2} – E_{NH3} \text{ [eV]}$$

3 Energy difference between stage A4 and A3 (same for B diagram):

$$E_{diff} = E_{A4} – E_{A3} \text{ [eV]}$$

4 Energy difference between stage A5 and A4 (same for B diagram):

$$E_{diff} = E_{A5} – E_{A4} + E_{H2O} \text{ [eV]}$$

5 Energy difference between stage A1 and A5 (same for B diagram):

$$E_{diff} = E_{A1} - E_{A5} + E_{N2} \text{ [eV]}$$

Energy difference between stages in mechanism with OH group on metal atom were calculated as follows:

1 Energy difference between stage C2 and C1 (same for D, E and F diagrams):

$$E_{diff} = E_{C2} - E_{C1} - E_{NO} - E_{NH3} \text{ [eV]}$$

2 Energy difference between stage C3 and C2 (same for D, E and F diagrams):

$$E_{diff} = E_{C3} - E_{C2} \text{ [eV]}$$

3 Energy difference between stage C4 and C3 (same for D, E and F diagrams):

$$E_{diff} = E_{C4} - E_{C3} + 2E_{H2O} \text{ [eV]}$$

4 Energy difference between stage C5 and C4 (same for D, E and F diagrams):

$$E_{diff} = E_{C5} - E_{C4} + E_{N2} \text{ [eV]}$$

The lowest energy systems were used for all the calculations.

Table S1 Energies for different structure and considered multiplicities.

(a)

Structure	Multiplicity	Energy [H]	Structure	Multiplicity	Energy [H]
A1	1	-15108.916	B1	1	-13195.657
	3	-15108.847		3	-13195.662
	5	-15108.722		5	-13195.535
	7	-15108.499		7	-13195.391
A2	1	-15238.233	B2	1	-13324.968
	3	-15238.201		3	-13324.956
	5	-15238.093		5	-13324.927
	7	-15237.958		7	-13324.833
A3	1	-15294.842	B3	1	-13381.577
	3	-15294.816		3	-13381.578
	5	-15294.691		5	-13381.506
	7	-15294.541		7	-13381.400
A4	1	-15294.836	B4	1	-13381.523
	3	-15294.724		3	-13381.545
	5	-15294.655		5	-13381.445
	7	-15294.577		7	-13381.405
A5	1	-15218.389	B5	1	-13305.087
	3	-15218.346		3	-13305.084
	5	-15218.239		5	-13305.051
	7	-15218.096		7	-13304.932

(b)

Structure	Multiplicity	Energy [H]	Structure	Multiplicity	Energy [H]
C1	1	-15184.068	D1	1	-13270.785
	3	-15184.060		3	-13270.780
	5	-15183.971		5	-13270.761
	7	-15183.816		7	-13270.650
C2	2	-15370.652	D2	2	-13457.303
	4	-15370.596		4	-13457.294
	6	-15370.476		6	-13457.216
	8	-15370.357		8	-13457.033
C3	2	-15370.723	D3	2	-13457.428
	4	-15370.630		4	-13457.432
	6	-15370.456		6	-13457.322
	8	not converged		8	-13457.173
C4	2	-15217.823	D4	2	-13304.560
	4	-15217.732		4	-13304.541
	6	-15217.537		6	-13304.396
	8	-15217.415		8	-13304.255
C5	2	-15108.273	D5	2	-13195.008
	4	-15108.190		4	-13195.003
	6	-15108.060		6	-13194.884
	8	-15107.838		8	-13194.712

(c)

Structure	Multiplicity	Energy [H]	Structure	Multiplicity	Energy [H]
E1	1	-15184.073	F1	1	-13270.798
	3	-15184.060		3	-13270.800
	5	-15183.956		5	-13270.780
	7	-15183.795		7	-13270.654
E2	2	-15370.652	F2	2	-13457.397
	4	-15370.591		4	-13457.338
	6	-15370.469		6	-13457.289
	8	-15370.293		8	-13457.105
E3	2	-15370.649	F3	2	-13457.444
	4	-15370.551		4	-13457.425
	6	-15370.463		6	-13457.281
	8	-15370.338		8	-13457.142
E4	2	-15217.809	F4	2	-13304.553
	4	-15217.597		4	-13304.530
	6	-15217.454		6	-13304.418
	8	-15217.728		8	-13304.256
E5	2	-15108.273	F5	2	-13195.008
	4	-15108.190		4	-13195.003
	6	-15108.060		6	-13194.884
	8	-15107.838		8	-13194.712
